# Learning debiased graph representations from the OMOP common data model for synthetic data generation

**DOI:** 10.1186/s12874-024-02257-8

**Published:** 2024-06-22

**Authors:** Nicolas Alexander Schulz, Jasmin Carus, Alexander Johannes Wiederhold, Ole Johanns, Frederik Peters, Natalie Rath, Katharina Rausch, Bernd Holleczek, Alexander Katalinic, the AI-CARE Working Group, Alice Nennecke, Henrik Kusche, Vera Heinrichs, Andrea Eberle, Sabine Luttmann, Khalid Abnaof, Soo-Zin Kim-Wanner, Heinz Handels, Sebastian Germer, Marco Halber, Martin Richter, Martin Pinnau, David Reiner, Jannik Schaaf, Holger Storf, Tobias Hartz, Nils Goeken, Janina Bösche, Alexandra Stein, Kerstin Weitmann, Wolfgang Hoffmann, Louisa Labohm, Christiane Rudolph, Christopher Gundler, Frank Ückert, Christopher Gundler

**Affiliations:** 1 Institute for Applied Medical Informatics, University Medical Center Hamburg-Eppendorf, Hamburg, Germany; 2 University Cancer Center Hamburg, University Medical Center Hamburg-Eppendorf, Hamburg, Germany; 3 Cancer Registry Hamburg, Hamburg, Germany; 4 Saarland Cancer Registry, Saarbrücken, Germany; 5 Cancer Registry Schleswig-Holstein, Lübeck, Germany

**Keywords:** Synthetic Data Generation, Standardized Electronic Health Records, Causal Discovery, Discrete Time Series, Structural Equation Models, Graphical Models, Constraint-based Causal Discovery, Gradient-Based Causal Discovery, DYNOTEARS, Temporal Association Rule Mining (TARM)

## Abstract

**Background:**

Generating synthetic patient data is crucial for medical research, but common approaches build up on black-box models which do not allow for expert verification or intervention. We propose a highly available method which enables synthetic data generation from real patient records in a privacy preserving and compliant fashion, is interpretable and allows for expert intervention.

**Methods:**

Our approach ties together two established tools in medical informatics, namely OMOP as a data standard for electronic health records and Synthea as a data synthetization method. For this study, data pipelines were built which extract data from OMOP, convert them into time series format, learn temporal rules by 2 statistical algorithms (Markov chain, TARM) and 3 algorithms of causal discovery (DYNOTEARS, J-PCMCI+, LiNGAM) and map the outputs into Synthea graphs. The graphs are evaluated quantitatively by their individual and relative complexity and qualitatively by medical experts.

**Results:**

The algorithms were found to learn qualitatively and quantitatively different graph representations. Whereas the Markov chain results in extremely large graphs, TARM, DYNOTEARS, and J-PCMCI+ were found to reduce the data dimension during learning. The MultiGroupDirect LiNGAM algorithm was found to not be applicable to the problem statement at hand.

**Conclusion:**

Only TARM and DYNOTEARS are practical algorithms for real-world data in this use case. As causal discovery is a method to debias purely statistical relationships, the gradient-based causal discovery algorithm DYNOTEARS was found to be most suitable.

**Supplementary Information:**

The online version contains supplementary material available at 10.1186/s12874-024-02257-8.

## Background

Synthetic data holds paramount importance in the medical domain, particularly concerning medical health records, due to its potential to circumvent critical challenges associated with data privacy and legal constraints. By generating synthetic data that mimics the statistical properties of real patient data, researchers and practitioners can conduct analyses and develop algorithms without directly accessing sensitive information, thus safeguarding patient privacy. Therefore, the utilization of synthetic data offers a promising avenue for advancing medical informatics research and innovation through data availability while upholding ethical standards and legal compliance specific to the healthcare sector [1–3].

In addition, the standardization of patient data is fundamental for research in the field of medical informatics. As data availability and interoperability in medicine are relatively underdeveloped compared to other sectors [4], the generation of standardized synthetic data has emerged as a crucial area of research. Even in instances where access to real patient data is granted, the availability of such data for large-scale and replicable analytics remains limited due to the commonly missing interoperability with other systems. Standardized synthetic data holds the promise to mitigate these challenges. In this context, the standardized electronic health record (EHR) format specified in the Observational Medical Outcomes Partnership (OMOP) Common Data Model (CDM), or OMOP CDM in short [5], is used in this research. Whereas OMOP CDM defines the database structure, specific vocabularies developed by the Observational Health Data Sciences and Informatics (OHDSI) initiative standardize the content of the database [6, 7].

Typical examples are cancer registries serving as repositories for monitoring and analyzing the epidemiological occurrences of cancer within populations. Found in numerous countries worldwide, these organizations offer invaluable insights into the trends, patterns, and disparities in cancer burden across different demographic groups and geographical regions. Despite their importance, cancer registries face several data-based challenges in their operations. Firstly, despite the richness of data, access to data by external researchers is commonly strictly regulated. Ensuring compliance with regulations concerning patient privacy and data protection is paramount to maintaining the integrity and trustworthiness of cancer registries as institutions. Moreover, the data housed within cancer registries are often prone to noise and inaccuracies, stemming from the inherent complexities of medical reporting. Consequently, robust methodologies deriving trustable synthetic data from their internal data sources appear attractive to allow research while preserving privacy.

Currently, black-box approaches grounded in deep learning methodologies are popular for deriving synthetic data [8–10]. However, despite their widespread adoption, these opaque models face notable challenges. Most importantly, opaque models do not make the data generation process explicit or interpretable. In other words, a physician is not able to understand the models’ assumptions that resulted in the synthetic data. However, understanding *how* the synthetic data is generated is crucial in medicine, as wrong assumptions about dependencies between symptoms and treatments can have fatal consequences. Consequently, the field of medical informatics requires the study of methods for generating synthetic data through explicit knowledge representations like graphs, which offer an alternative method with a focus on interpretability, verifiability, and intervention through human experts.

One approach to generating synthetic patient data through explicit knowledge representations is provided by Synthea [11]. Synthea builds its data generation process on so-called disease modules, which are graph representations of a disease and treatment progression over time. Each official graph is openly available on the GitHub repository [12] and can be inspected for a deeper understanding. By sampling from these graphs, life-long synthetic and standardized EHRs are generated. However, these graph representations are constructed by hand through a time-consuming process and thereby also subjective to the constructing expert and not generalizable between geographical regions [13]. The aim of this paper thus is to learn Synthea graphs in a data-driven fashion.

To achieve this, temporal rules are learnt from a real patient cohort and transferred into the Synthea graph structure. More precisely, the learnt temporal rules constitute the directed graph edges in the final Synthea graph. Hereby the temporal dimension of the learnt rules is crucial, as EHRs generated by Synthea span the whole lifetime of a patient.

However, learning meaningful temporal rules from real-world patient hospital encounter histories is difficult. From a statistical standpoint, these patient histories are highly confounded time series. For example, a basic medical treatment that is common to all patients regardless of their disease status like blood sampling or receiving a bandage can distort the whole learning process and hence the resulting graph. In the analysis pipeline the learnt rules become the direct edges in the Synthea graph, and the structure of the Synthea graph determines how the synthetic data is generated. Therefore, this study evaluates which algorithm learns the most qualitative and meaningful rules to eventually generate high-quality synthetic data. To achieve this, it is indispensable to learn debiased temporal rules rather than statistical if-then rules. Causal discovery is the sub-discipline of artificial intelligence (AI) which deals with learning cause-effect relationships from observational data and debiasing potential confounding variables from the dataset [14].

The aim of this paper thus is to learn Synthea disease modules in a data-driven fashion to have an interpretable, verifiable, and explicit knowledge representation that is at the same time scalable across institutions, patients, and diseases. To achieve this, a bridge is built and evaluated between the established tools of medical informatics, namely the OMOP format and Synthea graph representations. This is done by extracting relevant information from real-world patient records in OMOP format [15], learning temporal rules by statistical and causal AI algorithms, and representing the learned temporal rules as direct edges in a Synthea graph. The resulting graphs are evaluated quantitatively through common graph complexity measures and qualitatively by experts in the medical field. As the direct graph edges drive the potential data synthetization process, the evaluation process is focused on the graphs’ edges from multiple perspectives. We postulate that causal discovery algorithms can outperform simple statistical algorithms with regard to computational complexity, real-life applicability to high-dimensional data, and qualitative representation of the learned rules.

## Methods

**Fig. 1:**
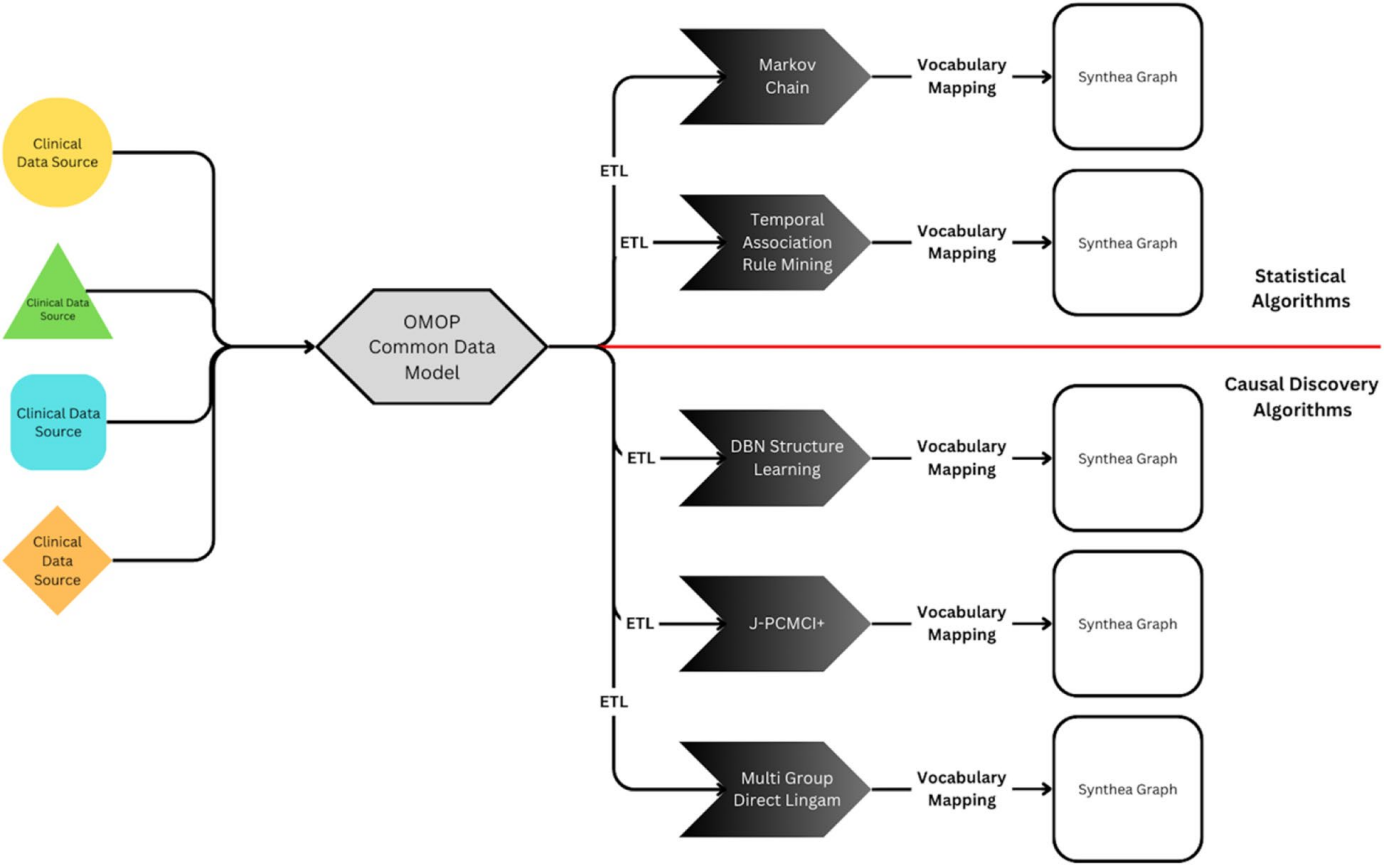
A schematic overview of the study design. The source data from a cancer registry was mapped into the standardized EHR format OMOP. Relevant data is extracted, transformed into a time series and analyzed by 2 statistical and 3 causal discovery algorithms, before each result is mapped into Synthea graph format. ETL: Extract, Transform, Load; OMOP: Observational Medical Outcomes Partnership

We postulate that combining Synthea, a widely used synthetic patient generator, with causal discovery algorithms and a standardized data model could significantly simplify the creation of high-quality and privacy-preserving synthetic data. An overview of the processes under research can be found in Fig. 1. After mapping the unstructured clinical source data into OMOP, five algorithms were implemented and evaluated to learn temporal rules from the patient's hospital encounter history. The details can be found in the following sections.

### Data

The anonymized data for this study was requested and retrieved from the cancer registry of Schleswig-Holstein, Germany. The dataset includes all adult patients living in the federal state of Schleswig-Holstein in Germany who were diagnosed with lung cancer in the period between 2016 and 2021. Given the non-standardized format of the raw data, we designed and implemented an Extract, Transform, Load (ETL) process for transforming the dataset into the OMOP [16] common data model. Accordingly, all subsequent parts of the analysis are independent of the syntactic and semantic properties of the original dataset.

Given the existing, standardized format for electronic health records, we designed additional ETL routes to transform the observational data included into a suitable time series format and into the algorithm used for analysis. Whereas the ETLs differ in detail depending on the downstream algorithm, the common grounds are covered in the following. Firstly, the observational data of the OMOP condition occurrence and the procedure occurrence table were grouped by patient identifier and ordered by time of encounter. Secondly, start and stop nodes were attached to the beginning and end of each time series respectively. In doing so, the computational models can learn which observations are more likely to occur at the beginning or the end of a patient history and it enables the following transformation into Synthea graphs, as start and stop nodes are required for sampling [17]. After extracting the relevant medical observations from the OMOP database and transforming them into time series, the learning algorithms are applied.

The discrete-time series was used to compute temporal rules with multiple algorithms of varying complexity. Each algorithm output defining temporal rules between two medical observations subsequently had to be mapped into the Synthea graph format as direct graph edges. The Synthea graph syntax is highly complex but well documented on their online resources [17]. In the context of this study, the relevant node type was ‘Encounter’ as defined by Synthea and the edge type used was ‘distributed transition’. These two were chosen to reduce complexity in automatic mapping from rules to edges, while still enabling subsequent synthetic data generation. In addition, the medical observations were mapped back from OMOP vocabulary to English free-text using data-specific concept relationships from the vocabulary database Athena [7].

### Algorithms

The algorithms evaluated in this study can be categorized into two separate groups, namely statistical approaches, and techniques of causal discovery. Whereas the algorithms in the statistical domain merely compute and extract observational statistical quantities, the algorithms belonging to the causal discovery domain are more complex. In essence, causal discovery algorithms claim to distinguish relationships within the data that are purely statistical and entail bias from cause-effect relationships, which are statistically debiased. Both groups, as well as the individual algorithms within each group, are presented in more detail below.

In the following, consider $\mathcal{N}$ independent realizations of discrete time series ${x}_{n,t}\in {\mathbb{C}}^{\mathcal{D}}$. An individual patient’s hospital history is denoted as ${x}_{n}$ and can vary on time axis $t\in \{0,.., T\}$ and the medical observations within each time step $d\in \mathcal{D}$.

#### Statistical approaches

##### Markov chain

In the first approach to computing Synthea graph edges from observational real-world EHR, the problem is defined as a Markov chain of first order. In other words, every medical observation is defined as a state, and the conditional probability of observing any other medical observation in the next step is calculated across patients. By incorporating the graph terminology of an antecedent and a consequent node in a directed graph edge, the transition probabilities are defined as:

$$P(consequent|antecedent)=\frac{\text{P}(\text{antecedent }\bigcap \text{ consequent})}{\text{P}(\text{antecedent})}$$

Thus, the conditional probability for each medical observation in ${x}_{n,t}$ to each medical observation in ${x}_{n,t+1}$ is calculated and averaged across the patient population $\mathcal{N}$.

##### Temporal Association Rule Mining

Temporal Association Rule Mining (TARM) mines temporal rules that are common to several sequences, i.e. time series. The implemented CMRules algorithm [18] identifies and outputs temporal rule according to two threshold values, namely sequential support (seqSup) and sequential confidence (seqConf) of a rule. Considering a temporal rule X $\to$ Y with any two random variables X,Y $\in \mathcal{D}$ these statistical thresholds are defined as:

$$seqSup(X\to Y) = \frac{sup(X Y)}{|S|}$$

$$seqConf(X\to Y) = \frac{sup(X Y)}{sup(X)}$$

where the notation *sup (X Y)* defines the number of observations where some set of medical observations X all occur before some other set of medical observations Y. The notation S defines the sequence database, meaning across all patient sequences $\mathcal{N}$.

##### Causal discovery algorithms

In statistics, it is common knowledge that association is not causation, but displaying temporal associations in a directed graph can easily be misinterpreted as such. In addition, if there is no method in place to filter out purely statistical relationships, the graph can become highly confounded. Therefore, learning a directed acyclic graph (DAG) from patient histories for interpretation by medical professionals needs to be debiased and display cause-effect relationships rather than if-then rules. Conceptually, a causal relationship is said to be present in X $\to Y$ if the random variable Y listens or responds to the presence of X [19].

##### Dynotears

DYNOTEARS or Dynamic NOTEARS is a Dynamic Bayesian Network Structure learning approach from data [20]. DYNOTEARS is a score-based optimization method falling into the category of gradient-based causal discovery approaches [21], which enables the application to high-dimensional real-world data. In this method, the observational data is structured in structural equations of endogenous and exogenous variables using a Structural Vector Autoregressive Model (SVAR). Afterwards, intra (${x}_{n,t} \to {x}_{n,t} )$ and inter-slice edges (${x}_{n,t} \to {x}_{n,t+1} )$ of the discrete time steps are identified and learnt using two matrices **W** and** A** for each group of links respectively. By elegantly reformulating the acyclicity constraint of the directed acyclic graph [22], the learning process can be defined as a continuous optimization problem.

##### J-PCMCI+

J-PCMCI+ is a constraint-based causal discovery algorithm, which extends the basic PCMCI algorithm [23] by learning inter and intra-slice edges from multiple multivariate time series by pooling [24]. The algorithm assumes causal sufficiency and employs conditional independence (CI) tests to identify causal relationships within the data. However, due to the constraint-based approach to the problem of causal discovery, the computational complexity of this algorithm grows drastically with the dataset size and dimensionality.

##### Multi Group Directed LiNGAM

The Multi Group Directed LiNGAM [25] is an algorithm that belongs to the group of functional causal models [21]. The algorithm extends the original Linear Non-Gaussian Acyclic Model (LiNGAM) [26] by jointly estimating shared causal relationships across datasets. This is done by estimating the shared causal ordering of variables through pairwise independence tests. Thereafter, causation and correlation are distinguished by approximating the consistency of associations through time and variable pairs.

#### Experimental design

To the best of the authors’ knowledge, no prior investigation has been done on how to learn symbolic graph representations for synthetic data generation (SDG) from real data while tying together the established standardized data formats and Synthea. Thus, the evaluation is approached from a quantitative as well as qualitative angle to provide foundations for further research.

#### Quantitative experiments

The quantitative experiments should give insights regarding the general applicability of the algorithms and the complexity of the resulting graphs. As real-world clinical patient data comes in varying sizes, it is crucial to investigate the algorithm’s robustness by manipulating data complexity. For that reason, we subsampled the full dataset into four different sizes. The detailed characteristics can be found in Table 1, where the first dataset corresponds to the full dataset and all others are random subsamples thereof. Each of the five models has been evaluated on all five datasets.

**Table 1:** An overview of the data samples used in this study. The average sequence length is the arithmetic mean of the length of all time series in the dataset and the dimensionality is the total amount of medical observations in the dataset

Data Set Number	Sample Size N	Average Sequence Length	Dimensionality
1	11641	5.487	726
2	5000	5.513	512
3	500	6.130	200
4	50	5.720	68
5	10	6.400	26

As previously discussed, the learned temporal rules of each model serve as directed edges in the final graphs. Each graph structure is assessed using six complexity measures, namely number of nodes, number of edges, graph density, average clustering, amount of strongly connected components, and flow hierarchy. Graph density reflects the ratio of actual edges to possible edges, indicating connectivity, ranging from 0 (sparse) to 1 (dense). Average clustering measures local interconnectedness, with values from 0 (no connection) to 1 (strong connection). Strongly connected components denote subgraphs where every pair of nodes has a directed path, with their quantity being the metric of interest. Flow hierarchy assesses node influence on information flow, ranging from 0 (equal influence) to 1 (hierarchical influence). These metrics therefore give insights into how each graph is structured without the need for display.

Finally, each graph is compared to every other graph in this research by the percentage of overlap with regard to their direct edges. In this way, the quantitative experiments answer questions of *how many,* and *which* rules are learned in comparison to other models and varying dataset sizes. In addition, the algorithm runtime is measured and incorporated in the evaluation process of each algorithm to provide insights into their practical applicability to real-world data.

### Qualitative evaluation

In addition to quantitative experiments, a qualitative evaluation was conducted to provide deeper insights into the learned graph representations. A digital questionnaire was designed specifically for this purpose, targeting experts from the medical domain, including individuals from German cancer registries and trained clinicians. Each expert was asked to assess two graphs, one graph based on statistical methods and one graph based on causal discovery with regard to interpretability, medical meaningfulness, and cause-effect relationships. In detail, the experts should indicate if

1. the graph appears interpretable,
2. the graph shows a consistent chronological order,
3. some of the edges make sense from a causal perspective,
4. and whether some of the edges do not make any sense on a Likert scale with five elements ranging from “strongly disagree” to “strongly agree”. The method used to generate the graphs was blinded.

## Results

### Quantitative results

**Fig. 2:**
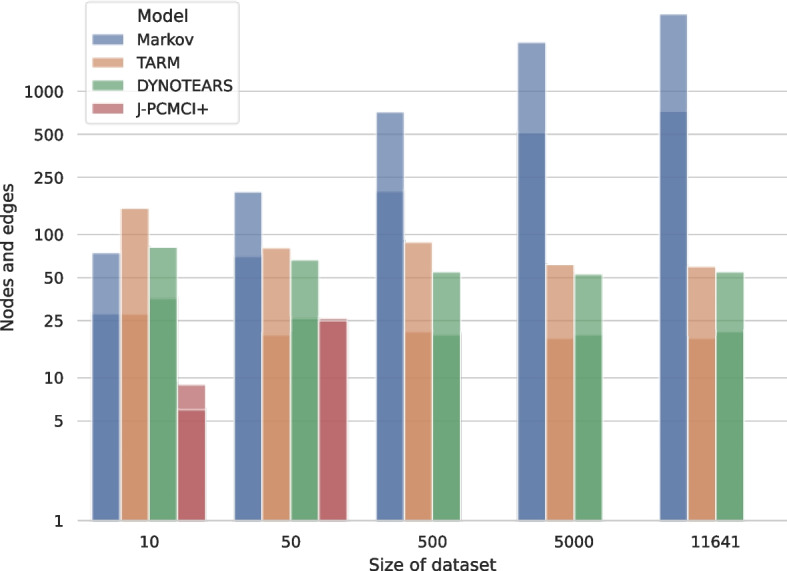
A comparison of the graph complexities in terms of nodes and edges whereas the latter value corresponds to the higher of the two bars. Whereas the Markov model learns graphs which grow exponentially with the dataset size, TARM and DYNOTEARS learn similarly sized graphs across all data sets

The resulting graph complexity measures are depicted per model across all 5 datasets in the Supplement (Tables S1-S4) and summarized in the following Figures. The main observation is that while the Markov chain *extracts* rules from the data, the other methods *learn* a compact set of rules that describe the data. The Markov model learns graph representations which grow exponentially with the data set size, however, the graph sizes for the other models stay consistent across increasing data set sizes (Fig. 2). In addition, the number of nodes and edges for TARM and DYNOTEARS are almost similar across all data sets. This results in immensely complex graphs for the Markov chain and graphs of humanly interpretable size for the other models.

**Fig. 3:**
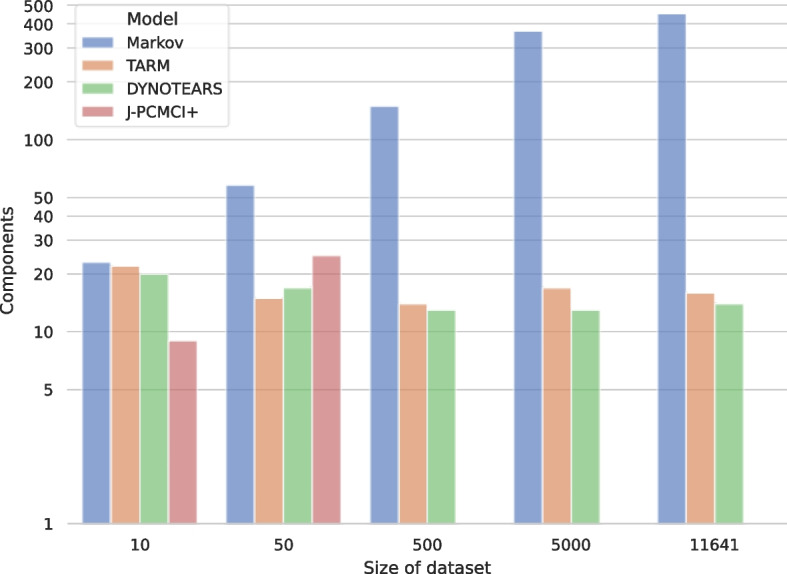
A comparison of the graph complexities in terms of strongly connected components. The amount of strongly connected components grows exponentially for the Markov model and stays consistent for TARM and DYNOTEARS

In directed graphs, the number of strongly connected components represent sets of nodes where every node is reachable from every other node within the same components, thereby providing insights into how information flows through the graph. Figure 3 compares the amount of strongly connected components across models and data set sizes. It can be observed that the amount of strongly connected components grows with the data set size for the Markov model and J-PCMCI+. The amount of strongly connected components does not increase for TARM and DYNOTEARS, however, also their graph complexity in terms of nodes and edges did not increase as seen in Fig. 2. As the amount of strongly connected components can maximally be the number of nodes in a graph, the ratio of strongly connected components to nodes stays consistent between models.

**Fig. 4:**
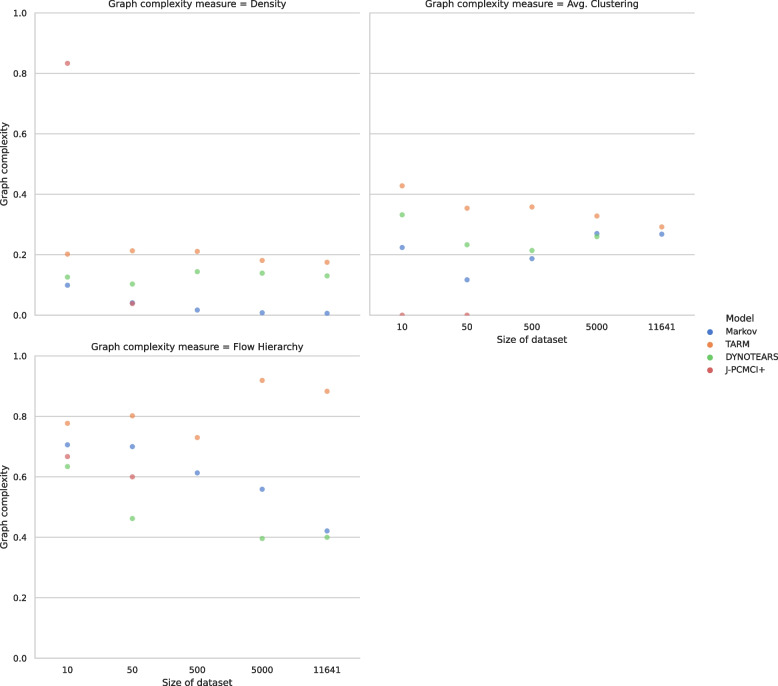
The comparison of different graph complexity measurements on different sizes of the dataset. For better visual comprehension, the high scores of DYNOTEARS for average clustering on the full dataset size and the flow hierarchy for the dataset with 500 samples are omitted. All values can be found in Tables S1-S4

The remaining three graph complexity measures are graph density, average clustering, and flow hierarchy depicted in Fig. 4. For the graph density, which provides a measure of connectivity, it is observed that the large Markov model graphs are the least dense overall. Whereas the J-PCMCI+ algorithm produced the densest graph on the smallest data set, it produced the less dense graph on the second smallest data set. Generally, the TARM model produced the densest or most connected graphs across all datasets and remained consistent across increasing data set sizes. In line with that, the average clustering coefficient, which is a measure of the local connectivity of nodes, is also observed to be the highest for TARM and closely followed by DYNOTEARS and then the Markov model. However, the average clustering coefficient for J-PCMCI+ graphs is zero. Finally, the flow hierarchy is also highest for TARM. For this metric, however, DYNOTEARS produced the lowest flow hierarchy scores across data sizes.

**Fig. 5:**
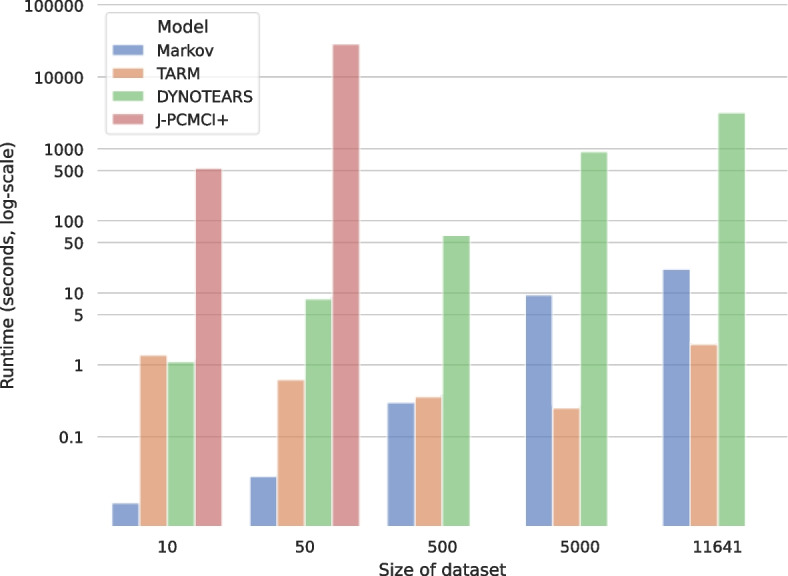
Visualization of the algorithm runtimes in seconds per dataset. Whereas the two statistical algorithms (Markov and TARM) and the gradient-based causal discovery algorithm (DYNOTEARS) are similarly performant, the constraint-based causal discovery algorithm (J-PCMCI+) displays dissimilar computational complexity. The values for each algorithm are displayed in Table S5

As aforementioned, J-PCMCI+ could only be applied to the two smallest data sizes. Figure 5 highlights the issue, as the computation time greatly exceeds all previous approaches already on the second smallest dataset. Whereas the algorithm runtime for the second smallest dataset was a matter of few seconds for Markov, TARM, and DYNOTEARS, J-PCMCI+ needed almost eight hours to complete on the same computational resources. In a similar way, Multi Group Direct LiNGAM was found to generally not apply to this use case as the smaller dataset violated the data requirement $\mathcal{N}>\mathcal{D}$ and the larger datasets which fulfill this requirement were too high-dimensional for the algorithm to finish in reasonable time.

**Fig. 6:**
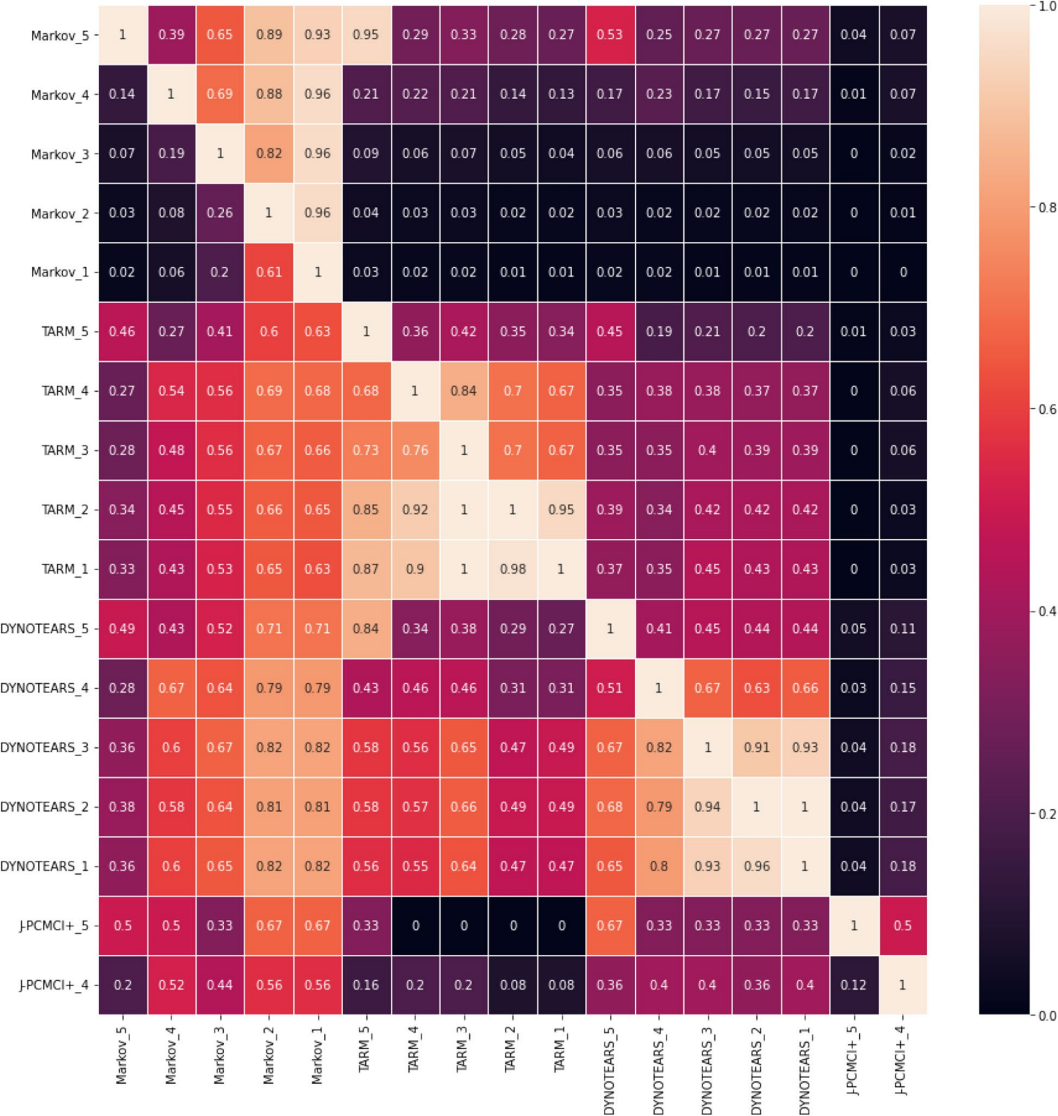
The adjacency matrix displaying the percentage of identical edges between all graphs in the study as a heatmap. Rows and columns are named after the format ‘Algorithm’ + ‘Dataset Number’. A cell describes how much the graph specified in the row overlaps with the graph specified in the column

Figure 6 is a display of similarity measures comparing the intersection of any two graphs in the dataset. The measure of similarity is the percentage of identical directed edges of any two graphs of the form X $\to Y$, disregarding the edge weight. The Markov model on the complete dataset has the largest number of rules and is represented in any other graph to the largest extent. The J-PCMCI+ graphs have the smallest intersection with the other graphs in the experiment. Each algorithm, DYNOTEARS as well as the TARM approach learn exactly the same graph for the data with 5000 samples as the for the complete dataset. However, comparing the graphs of the two models shows an overlap of 42-49% maximally.

### Qualitative results

The qualitative assessment, based on the feedback from eight domain experts, illuminated both the potential and the challenges associated with the automatically derived graphs (Supplement SF1-SF3). Drawing on the quantitative findings previously reported, we chose TARM as the statistical method and DYNOTEARS as the causal discovery method for evaluation.

After three obtained evaluations, feedback from the experts cast doubts on our initial evaluation procedure. Notably, experts expressed difficulty in providing feedback due to the uncommon usage of generated graphs within cancer registries and reported concerns regarding the reliability due to the absence of a gold standard for comparison. Responding to this feedback, we revised the evaluation questionnaire and introduced the visualization of a “gold standard“ module, featuring a manually constructed graph based on literature findings for lung cancer, integrated within the software by default. For consistency, we only report the results from the revised questionnaire.

**Fig. 7:**
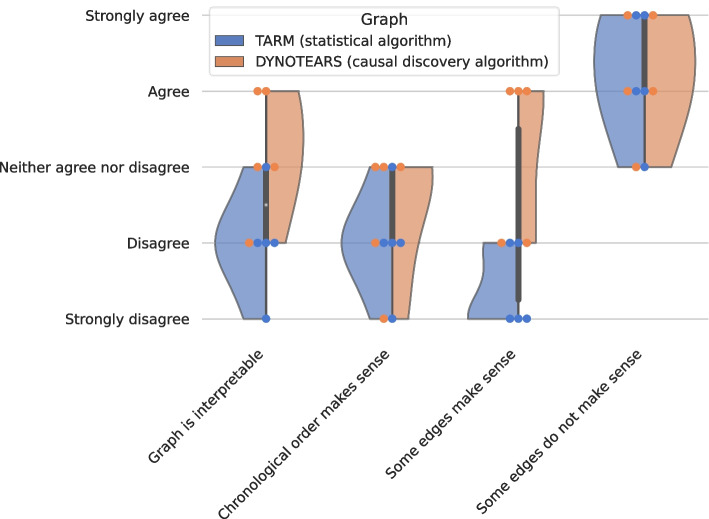
The reported values for the four statements which should be specified on the Likert scales with five elements. According to the experts, both graphs contain edges which do not make sense semantically. In the other statements, the graph generated by the causal discovery algorithm obtains better ratings

Figure 7 visually illustrates the feedback, indicating that the quality of automatically generated graphs lags behind that of hand-made graphs, while the graph generated by the causal algorithm was preferred. While both graphs were deemed to contain some edges with limited semantic sense, the graph produced by the causal discovery algorithm outperformed its counterpart in all other statements, with experts rating its edges as more meaningful and interpretable overall.

When asked for a summary, 80% of experts observed that the graph generated by TARM contained the highest number of nonsensical edges. Additionally, 60% of experts suggested that the graphs could be useful for guiding manual graph construction. Consensus among experts highlighted the hand-derived graph from Synthea as the most interpretable and possessing the most reliable rules.

Notably, the free-text responses provided insights beyond the scope of the research questions. In statements regarding the general usability of the graphs for representing patient therapies, some general criticism of the overall data structure was raised. As an example, one expert mentioned the need for additional metadata beyond the graph structure, for example regarding the intention of therapy (palliative or curative), to enhance the relevance and accuracy of the generated graphs in capturing clinical dynamics relevant for cancer registries.

## Discussion

Processing clinical data in general and cancer registry data, in particular, presents a challenge due to the magnitude of noise and complexity inherent in medical records. The abundance of variables and the intricate interplay between them leads to complexity and makes extracting meaningful insights a challenging task.

While this complexity poses challenges for most of the compared algorithms, it contains potential for causal discovery, too. Causal discovery appears important for this use case as it may debias the downstream graph representation from purely statistical relationships, as simply extracting statistical quantities will lead to meaningless rules. For example, medical observations which occur independent of disease progression of the patient (e.g. blood sampling) will create a bottleneck in the resulting graph as all other medical observations prior would be displayed to lead to blood sampling. As it may be correct that a lot of medical observations are followed by blood sampling, displaying it as a bottleneck rather than a reoccurring observation is strongly biased. Since any medical observation can only occur once in a Synthea graph, however, this is the only way to display these statistical rules and highlights the need for a causal method which can distinguish which random variable responds to the presence of another in the causal ordering.

On the one hand, the experiments regarding varying dataset sizes unveiled robustness issues for most of the applied algorithms. Whereas the Markov chain is not able to break down a high-dimensional dataset into a compact set of rules, J-PCMCI+ and Direct LiNGAM are practically not applicable to high-dimensional data due to computational complexity. Only TARM and DYNOTEARS were able to learn a compact set of temporal rules from the full dataset containing all lung cancer patients. On the other hand, despite learning a similarly sized graph representation from high-dimensional data, TARM and DYNOTEARS graphs displayed a maximum of 42-49% on the similarity measure. This strongly suggests that the causal discovery algorithm DYNOTEARS indeed learns qualitatively different rules compared to the basic statistical TARM and can debias the graph. This observation is additionally supported by the qualitative evaluation, where experts assign higher scores regarding the reliability of the causal approach.

During the experiment, we observed that each algorithm resulted in graphs which are of different size and shape. Likely, these differences are due to the way in which rules are learned from patient trajectories by each algorithm. As the goal was to create an output as close to the original Synthea graph as possible, a highly hierarchical and connected graph representation is desired. The hierarchical structure of a graph is crucial for interpreting the nodes and edges as event sequences on a temporal dimension. However, since the flow hierarchy within the learned graphs is intrinsic to the collection of acquired rules and cannot be modified during post-processing, the data-driven graphs are likely to have encountered challenges during qualitative assessment from experts. Especially DYNOTEARS graphs, which learned lagged causal relationships from the patient trajectories were likely to suffer from this. As Synthea graphs do not explicitly display a time dimension, but rather do so implicitly through their hierarchy, DYNOTEARS likely suffered from information loss by mapping it into Synthea graphs. However, the experts considered the resulting output as already sufficient for initially supporting the creation of the graphs.

The presented study is not without limitations. First, the methods extracting direct causal relations are currently not able to estimate the more complex operations that Synthea offers, such as conditional statements or loops. Future research could explore these aspects to generate more expressive and accurate graph structures. Second, the inclusion of more medical experts would additionally improve the power of the qualitative analysis. Finally, the visualization itself appears to matter significantly. Given the comments of the qualitative analysis, the best grades for interpretability for the human-made graph result not only from its semantically meaningfulness but from the additional meaningful hierarchical order of the nodes. Accordingly, including this additional information requires further research.

In summary, while technology has not yet advanced to autonomously generate accurate graphs representing patient trajectories, it can serve as a valuable support tool for humans, enhancing their decision-making processes based on existing literature and insights extracted from the data.

## Conclusions

Learning symbolic representations for synthetic data appears as a promising option to mitigate challenges associated with the usage of clinical data. Synthesizing patient data from explicit representations is a non-negotiable requirement in medicine, as it is a central verifiable method for a real-world problem statement without a ground truth dataset. However, learning unsupervised graph representation from real patient histories is a task prone to statistical bias. Causal discovery can provide a solution to this issue, but most approaches are not scalable to high dimensions. Within the direct comparison of statistical approaches and causal discovery approaches, gradient-based causal discovery was found to be the most suitable approach. By adopting the required processes to a common data model like OMOP, the obtained results are utilizable for other types of clinical conditions, too. Accordingly, while the method might not be ripe for an unsupervised extraction of the governing rules, it holds the potential to assist human experts in creating verifiable knowledge bases for synthetic data.

### Supplementary Information

Supplementary Material 1.

Supplementary Material 2.

Supplementary Material 3.

Supplementary Material 4.

Supplementary Material 5.

Supplementary Material 6.

Supplementary Material 7.

## Data Availability

The raw data is available on reasonable request and after the corresponding clearance process from the cancer registry in Schleswig-Holstein. The scripts for generating the OMOP tables and running analysis will be made publicly available under a permissive open-source license after acceptance. All reported values are publicly available in the Supplement.
