# Supplementary material for: Learning debiased graph representations from the OMOP common data model for synthetic data generation
Source: BMC Med Res Methodol. 2024 Jun 22;24:136. doi: 10.1186/s12874-024-02257-8 (PMC11193245; doi:10.1186/s12874-024-02257-8)
Supplement: Supplementary file 1 — Supplementary Material 1. [file 12874_2024_2257_MOESM1_ESM.docx]

Supplementary Material

**Learning Debiased Graph Representations from the OMOP Common Data Model for Synthetic Data Generation**

by Nicolas Alexander Schulz, Jasmin Carus, Alexander Johannes Wiederhold, Ole Johanns, Frederik Peters, Natalie Rath, Katharina Rausch, Bernd Holleczek, Alexander Katalinic, the AI-CARE Working Group, and Christopher Gundler

**Characteristics of the different graphs**

**Table S1: Markov chain**

| Data Set Number | Nodes | Edges | Density | Average Clustering | Components | Flow Hierarchy |
| --- | --- | --- | --- | --- | --- | --- |
| 1 | 725 | 3458 | 0.006 | 0.268 | 453 | 0.421 |
| 2 | 513 | 2203 | 0.008 | 0.270 | 367 | 0.559 |
| 3 | 201 | 714 | 0.017 | 0.187 | 150 | 0.613 |
| 4 | 70 | 200 | 0.041 | 0.117 | 58 | 0.700 |
| 5 | 28 | 75 | 0.099 | 0.224 | 23 | 0.706 |

**Table S2: Temporal Association Rule Mining (TARM)**

| Data Set Number | Nodes | Edges | Density | Average Clustering | Components | Flow Hierarchy |
| --- | --- | --- | --- | --- | --- | --- |
| 1 | 19 | 60 | 0.175 | 0.292 | 16 | 0.883 |
| 2 | 19 | 62 | 0.181 | 0.328 | 17 | 0.919 |
| 3 | 21 | 89 | 0.211 | 0.358 | 14 | 0.730 |
| 4 | 20 | 81 | 0.213 | 0.354 | 15 | 0.802 |
| 5 | 28 | 153 | 0.202 | 0.428 | 22 | 0.777 |

**Table S3: DYNOTEARS**

| Data Set Number | Nodes | Edges | Density | Average Clustering | Components | Flow Hierarchy |
| --- | --- | --- | --- | --- | --- | --- |
| 1 | 21 | 55 | 0.130 | 0.460 | 14 | 0.400 |
| 2 | 20 | 53 | 0.139 | 0.260 | 13 | 0.396 |
| 3 | 20 | 55 | 0.144 | 0.214 | 13 | 0.454 |
| 4 | 26 | 67 | 0.103 | 0.233 | 17 | 0.462 |
| 5 | 36 | 82 | 0.126 | 0.332 | 20 | 0.634 |

**Table S4: J-PCMCI+**

| Data Set Number | Nodes | Edges | Density | Average Clustering | Components | Flow Hierarchy |
| --- | --- | --- | --- | --- | --- | --- |
| 1 | / | / | / | / | / | / |
| 2 | / | / | / | / | / | / |
| 3 | / | / | / | / | / | / |
| 4 | 26 | 25 | 0.0384 | 0.000 | 25 | 0.600 |
| 5 | 9 | 6 | 0.8333 | 0.000 | 9 | 0.667 |

## **Table S5: Runtime per Algorithm in Seconds**

| Data Set Number | Markov chain | TARM | DYNOTEARS | J-PCMCI+ | Multi LiNGAM |
| --- | --- | --- | --- | --- | --- |
| 5 | 0.0122 | 1.377 | 1.1098 | 542.5946 | / |
| 4 | 0.0283 | 0.627 | 8.2777 | 28428.437 | / |
| 3 | 0.3013 | 0.364 | 63.0734 | / | / |
| 2 | 9.3957 | 0.255 | 921.249 | / | / |
| 1 | 21.8964 | 1.933 | 3201.210 | / | / |
