# Supplementary figures and images for: Learning debiased graph representations from the OMOP common data model for synthetic data generation
Source: BMC Med Res Methodol. 2024 Jun 22;24:136. doi: 10.1186/s12874-024-02257-8 (PMC11193245; doi:10.1186/s12874-024-02257-8)

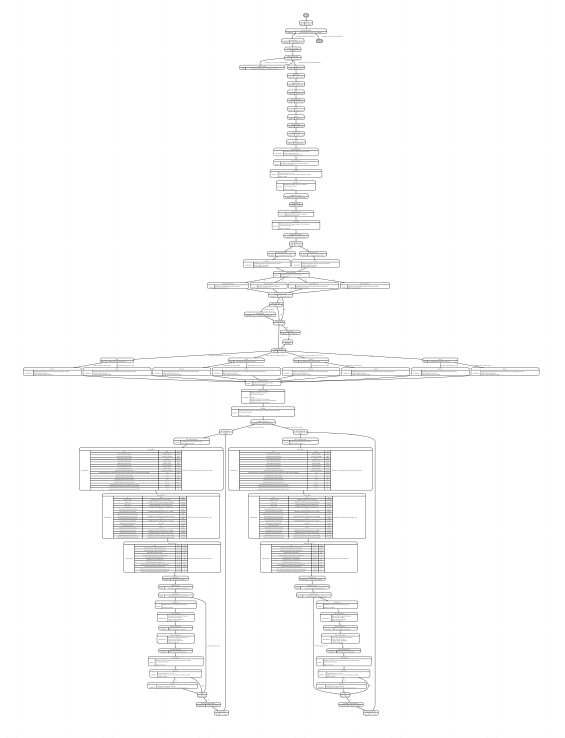

Supplement: Supplementary file 2 — Supplementary Material 2. [file 12874_2024_2257_MOESM2_ESM.png]

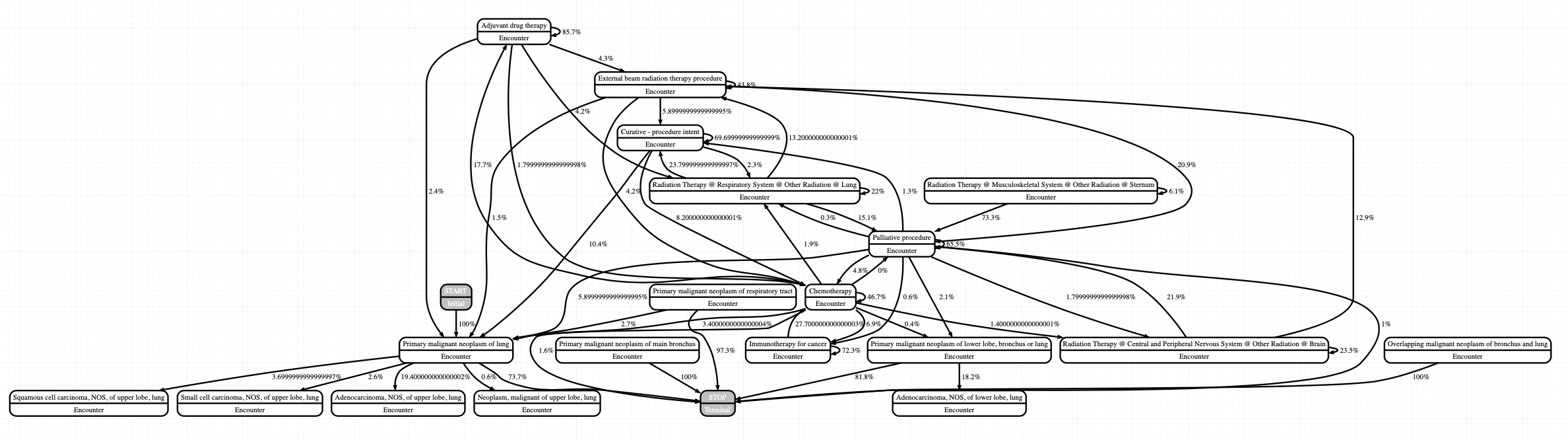

Supplement: Supplementary file 4 — Supplementary Material 4. [file 12874_2024_2257_MOESM4_ESM.png]

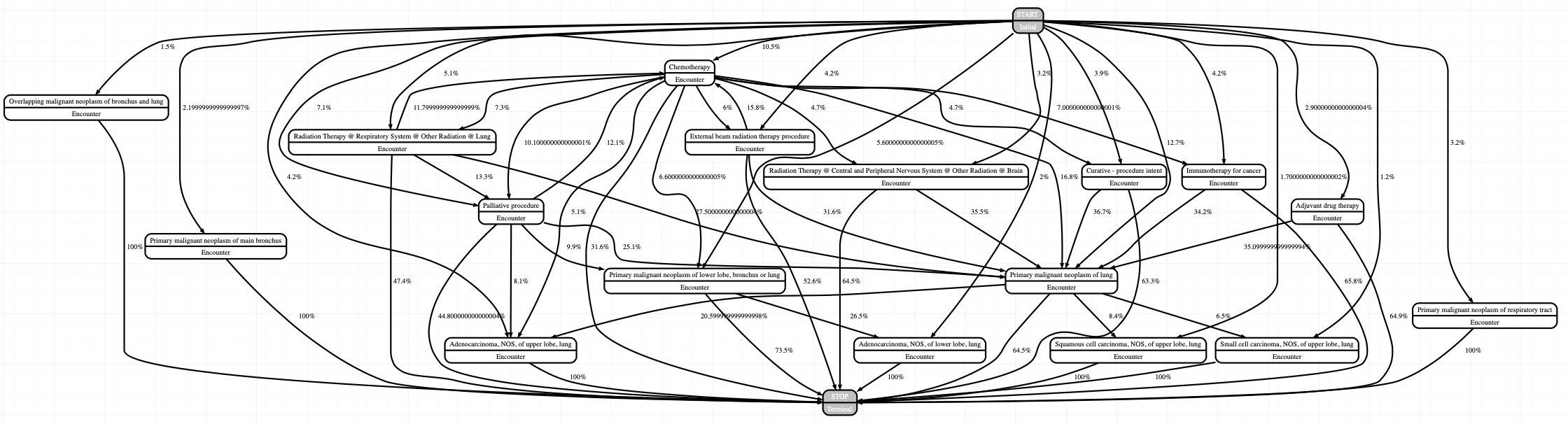

Supplement: Supplementary file 5 — Supplementary Material 5. [file 12874_2024_2257_MOESM5_ESM.png]
